# α6 Integrin and CD44 Enrich for a Primary Keratinocyte Population That Displays Resistance to UV-Induced Apoptosis

**DOI:** 10.1371/journal.pone.0046968

**Published:** 2012-10-10

**Authors:** Helen Wray, Ian C. Mackenzie, Alan Storey, Harshad Navsaria

**Affiliations:** 1 Blizard Institute of Cell and Molecular Science, Queen Mary’s School of Medicine and Dentistry, Whitechapel, London, United Kingdom; 2 Department of Molecular Oncology, Weatherall Institute of Molecular Medicine (WIMM), University of Oxford, John Radcliffe Hospital, Oxford, United Kingdom; University of Birmingham, United Kingdom

## Abstract

Epidermal human keratinocytes are exposed to a wide range of environmental genotoxic insults, including the UV component of solar radiation. Epidermal homeostasis in response to cellular or tissue damage is maintained by a population of keratinocyte stem cells (KSC) that reside in the basal layer of the epithelium. Using cell sorting based on cell-surface markers, we have identified a novel α6 integrin^high+^/CD44^+^ sub-population of basal keratinocytes. These α6 integrin^high+^/CD44^+^ keratinocytes have both high proliferative potential, form colonies in culture that have characteristics of holoclones and have a unique pattern of resistance to apoptosis induced by UVB radiation or by agents that induce single- or double strand DNA breaks. Resistance to UVB induced apoptosis in the α6 integrin^high+^/CD44^+^ cells involved increased expression of TAp63 and was overcome by PI-3 kinase inhibition. In marked contrast, the α6 integrin^high+^/CD44^+^ cells were sensitive to apoptosis induced by the cross-linking agent cisplatin, and imatinib inhibition of c-Abl blocked the ability of cisplatin to kill α6 integrin^high+^/CD44^+^ cells. Our findings reveal a population of basal keratinocytes with long-term proliferative properties that display specific patterns of apoptotic resistance that is dependent upon the genotoxic stimulus, and provide insights into how these cells can be targeted with chemotherapeutic agents.

## Introduction

The human epidermis is a stratified epithelium that maintains its integrity through a process of constant regeneration, driven by a population of keratinocyte stem cells (KSCs) in the basal layer [1]. The search to identify human KSCs has focused on the principle of adhesion to the basement membrane. *In vitro* it has been shown that the keratinocytes that rapidly adhere to cell culture plates form tightly packed colonies, termed holoclones, which have the greatest long-term growth potential and so are likely to contain stem cells [2], [3]. β1 integrin and α6 integrin expression is found on the basal surface of basal layer keratinocytes *in vivo* and identifies cells with a high growth potential *in vitro* [4], [5]. Malignancies display the common characteristic of excessive proliferation, suggesting that tumour growth is driven by a subset of malignant stem cells [6], [7]. The cell adhesion receptor CD44 has been shown to be expressed on the surface of tumour initiating cells in solid tumours of the breast [8], pancreas [9], prostate [10] and squamous cell carcinomas of the head and neck (SCCHN) [11]. In vitro, CD44-positive tumour cells display self-renewal properties indicative of stem cell behaviour [12]. Whilst the expression of CD44 in the epidermis has been confirmed [13], this surface protein is currently not thought to play a role in driving keratinocyte cell growth.

The epidermis is frequently exposed to sunlight, with the ultra-violet B (UVB) fraction of the radiation able to penetrate the surface of the epidermis, directly damaging the DNA of the keratinocytes via formation of cyclobutane pyrimidine dimers (CPDs) and 6–4 photoproducts [14]. The response of the keratinocytes to DNA damage is to undergo cell cycle arrest. If the damage is repairable via the nucleotide excision repair complex (NER) [15] the cell returns to the normal cell cycle. If DNA damage repair is not possible, the cell undergoes programmed cell death [16]. Following the shedding of apoptotic ‘sunburn’ cells from the surface, the epidermis repopulates to maintain the stratified epidermal barrier, indicating a sub-population of cells survives apoptotic induction.

In cancer cell lines CD44-positive cells have been shown to display a resistance to apoptosis following treatment with chemotherapy agents [17]. The aim of this study is to identify if there is a population of basal human keratinocytes that are positive for expression of CD44 and display an altered apoptotic response compared to the rest of the cell population. Here we report that a fraction of keratinocytes with the α6 integrin^high+^/CD44^+^ phenotype display a high growth potential, are able to resist cell death following UVB irradiation through high expression of TAp63 and activation of the PI3-kinase pathway, yet are sensitive to cell death induced by the cisplatin-activated c-abl pathway.

## Materials and Methods

### Cell Culture

Primary human keratinocytes were isolated from redundant facelift skin and passage 2–3 cells were used in all experiments unless otherwise stated. Keratinocytes were grown on mitomycin-treated 3T3 feeder layers in growth factor-containing medium (DMEM-F12 modified medium, PAA, Yeovil, UK) supplemented with 10% foetal bovine serum (FBS) (Biowest, East Sussex, UK), 1% (vol/vol) glutamine, 5 µg/ml insulin, 0.4 µg/ml hydrocortisone, 5 µg/ml transferrin, 10^−10^ M cholera toxin, 0.013 µg/µl lyothyronine (Sigma, Poole, UK) and 10 ng/ml EGF (Serotec, Oxford, UK) at 37°C in 10% CO_2_.

### Immunofluorescence

Frozen skin sections and primary human keratinocytes were fixed with 4% paraformaldehyde for 10 min at room temperature, washed with PBS, permeablised by incubating with 0.2% (v/v) Triton X in PBS for 5 min at RT if required and blocked with 5% donkey serum (Sigma, Poole, UK) at room temperature for 1 hour. Primary antibodies against human α-6 integrin (CD49f, BD Bioscience, Oxford, UK), human CD44 (AbD Serotec, Oxford, UK) or human total p63 (Santa Cruz Biotechnology, Heidelberg, Germany) were incubated at 4°C overnight with a further 3 washes completed before incubation with the relevant donkey secondary antibodies (Molecular Probes, Paisley, UK) at room temperature for 1 hour. Cells were then mounted with coverslips using Vectashield-DAPI mounting medium (Vectorlabs, Petersfield, UK) and analysed using a Nikon Eclipse TC2000S microscope (Nikon, Kingston, UK).

### Flow Cytometry

Cells were grown at clonal density until they reached 60% confluence (5 days), washed with PBS and removed from the culture dish using trypsin-EDTA (PAA, Yeovil, UK). Following neutralisation and washing with PBS the cells were resuspended at 1×10^6^/ml in PBS/1% FBS with 5×10^5^ cells used per incubation and analysis. Incubation was at 4°C for 30 min using CD44-FITC and CD49f-PE-Cy5 conjugated antibodies (BD Bioscience, Oxford, UK). Simultaneously cells were incubated with the corresponding IgG isotype controls (BD Bioscience, Oxford, UK). Cells were then washed in PBS/1% FBS and either recorded as live cells on a BD LSRII FACS machine or fixed cells following treatment with 1% paraformaldehyde. FACSDiva software was used to analyse all samples, with isotype and single-colour negative controls used to establish compensation settings, and a minimum of 30,000 events recorded per sample. For sorting of the cells a BD FACSAria machine was used. Other live cell-surface flow cytometry analysis used the BD antibodies β1 integrin (CD29-APC), transferrin receptor (CD71) with biotynilated-PE secondary, and appropriate IgG isotype controls (BD Bioscience, Oxford, UK). The FITC, PE and PE-Cy5 antibodies were excited using an argon laser (488 nm) and collected using a 530/30 nm filter (FITC), 575/26 nm filter (PE), and 675/20 nm filter (PE-Cy5). The APC antibody was excited using the 633 nm red diode and collected using a 660/20 nm filter.

### Cell Proliferation Assay

To determine the proliferative capacity *in vitro* of sub-populations of keratinocytes, cells were fractionated on the basis of their cell surface phenotype and grown in a long-term culture experiment. Following sorting, the sub-populations were seeded out at an initial density of 5×10^3^ cells per well of a 24-well plate onto a 3T3 feeder layer. In addition the relative ‘remainder’ fractions and total un-fractionated cell population (UF) were plated. All fractions per experiment were plated in triplicate, carried in parallel and passaged at the same time. At first passage all cells from each fraction were pooled and plated equally into three wells of a 6-well plate. At subsequent passages, cells from each well were counted and re-plated at 5×10^4^ cells per well of a 6-well plate, irrespective of cell yield. Serial passaging continued until each fraction had been grown to exhaustion. The cumulative total cell output for the initial fraction (5×10^3^ cells) was calculated assuming that all cells had been replated at each passage. Total cell output per original fractionated cell in each population was then calculated.

### Apoptosis Induction

Cells plated in dishes were grown to 50% confluence (4 days) and washed in PBS. A thin layer of PBS was added prior to UVB irradiation at increasing doses from 1 to 40 mJ/cm^2^. UVB irradiation was from a UVP Multiple Ray Lamp (Ultra-violet Products, Cambridge, UK) (MRL-58 model) fitted with F8T5 bulbs producing a sharp emission at 312 nm corresponding to mid-range UVB, calibrated before each experiment with a UVX Radiometer (UVP, Cambridge, UK). The PBS was then replaced with medium and cells grown for a further 6 to 48 hours dependent upon apoptosis assay. Chemical induction involved addition of chemotherapeutic drugs to the cell culture medium at increasing final doses; Etoposide 10–100 µg/ml, Cisplatin 25–200 µg/ml, Camptothecin 0.5–4 µM and Bleomycin 25–200 µg/ml (Sigma, Poole, UK) for 24 to 72 hours. The PI-3 kinase inhibitors Wortmannin (1 µM) and LY294002 (50 µM) and the tyrosine kinase inhibitor imatinib (1 µM) were added 2 hr prior to apoptosis induction. Doses of all drugs were chosen following preliminary dose-response assays, IC50 values of the drugs and previous relevant studies.

### Apoptosis Detection

Live cells were analysed for Annexin-V binding (Annexin V-FITC antibody, BD Bioscience, Oxford, UK) using the cell surface staining method described above followed by an incubation with the antibody in Annexin-V Binding Buffer (BD Bioscience, Oxford, UK) for 15 min at room temperature, a wash with PBS and the addition of 200 ng/ml DAPI directly prior to flow cytometry analysis to distinguish non-viable cells.

For Caspase-3 detection cells grown in 6-well plates were fixed with 4% paraformaldehyde, washed with PBS and permeablised with 0.2% Triton-X 100 in PBS for 5 min at room temperature. Cells were blocked using 5% goat serum/0.1% Tween 20 in PBS for 2 hours and washed ×1 in PBS. Rabbit anti-human Anti-Active Caspase-3 (Promega, Southampton, UK) was incubated at 1∶250 in 5% goat serum/1% Tween 20/PBS overnight at 4°C. Cells were washed in PBS (10 min×2), in 1% Tween 20/PBS (10 min×2) and in PBS (10 min×2). The 2° antibody Alexa Fluor 488 goat anti-rabbit IgG (H+L) (Invitrogen) was incubated 1∶500 in PBS for 2 hours at room temperature. Cells were washed in PBS (5 min×2), 1% Tween 20/PBS (5 min×1) and PBS (5 min×1). Cells were mounted using DAPI mounting medium and coverslips with fluorescence viewed using the Nikon Eclipse TE 2000-S microscope.

Cells analysed for TdT-mediated dUTP nick end-labelling (TUNEL) were removed from the dish, incubated with cell surface antibodies and fixed overnight as described earlier. Cells were then washed in PBS (x2), incubated in TUNEL (Promega) at 37°C for 1 hour, washed in PBS x1 and Triton-X 100 (5 min x1) before addition of 1 µg/ml DAPI in PBS 30 min before flow cytometry analysis.

### QRT-PCR

Total RNA was extracted from keratinocytes using the RNeasy Micro Kit (Qiagen, West Sussex, UK) according to manufacturer’s instructions. cDNA was generated using the SuperScript III First-Strand Synthesis SuperMix Kit (Invitrogen, Paisley, UK). Positive controls were transcribed from total human RNA (Stratagene QPCR Human Reference Total RNA, Agilent Technologies UK Limited, Stockport, UK) and negative controls with the omission of sample RNA (no template control, NTC) or omission of RT Enzyme Mix (no reverse transcriptase control, -RT). qRT/PCR was performed with Syber Green PCR master mix (Applied Biosystems, Warrington, UK) and results analysed using 7500 System Software (Applied Biosystems). The reaction was run at 95°C for 10 min followed by 45 cycles at 95°C for 15 s, 60°C for 30 s and 72°C for 40 sec and then one cycle at 95°C for 1 min. Forward (3′ to 5′) and reverse (5′ to 3′) primers were designed for ΔNp63 (Forward GGAAAACAATGCCCAGACTC, Reverse GTGGAATACGTCCAGGTGGC) and TAp63 (Forward AAGATGGTGCGACAAACAAG, Reverse AGAGAGCATCGAAGGTGGAG). Quantification was obtained by normalising the target genes against the GAPDH (Forward CACCCAGAAGACTGTGGATGG, Reverse GTCTACATGGCAACTGTGAGG) or 28s-r-RNA (Forward GCCGGGGGCCTCCCACTTAT, Reverse TGGCGGAATCAGCGGGGAAA) housekeeping genes.

## Results

### High Expression of CD44 and α6 Integrin Identifies a Subset of Human Keratinocytes with a High Proliferative Capacity *in vitro*

**Figure 1:**
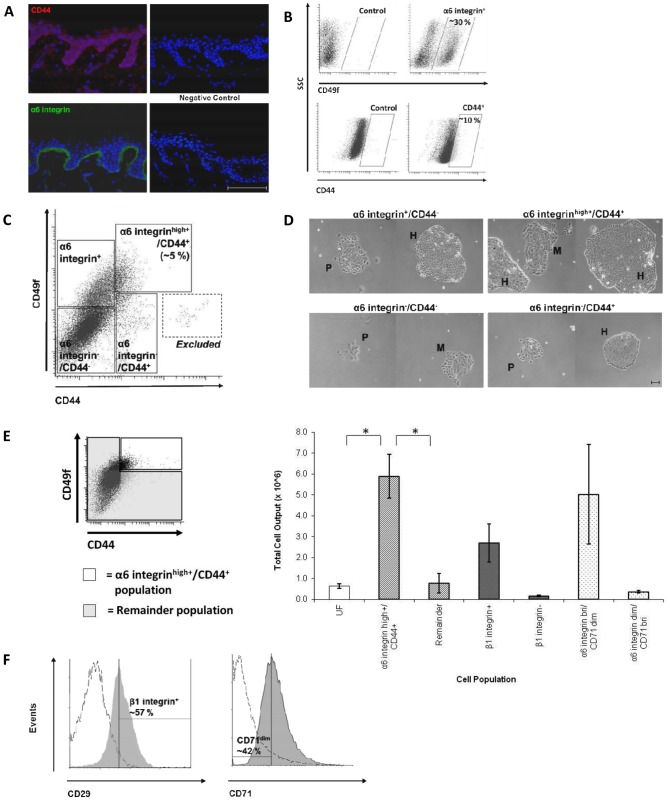
α6 integrin and CD44 identify a subset of human keratinocytes with long-term growth properties. (A) Immunofluorescence histochemistry of frozen normal human skin probed with an antibody against CD44 (red) or α6 integrin (green). Negative controls probed with blue nuclear stain (DAPI) only. Representative images of n = 3; scale bar = 100 µm. (B) Primary keratinocytes analysed for expression of α6 integrin using flow cytometry showed an average of ∼30% α6 integrin^+^ cells and ∼10% CD44^+^ cells (n = 5). (C) Primary keratinocytes analysed for co-expression of both α6 integrin and CD44 using FACS Diva software. The CD44^+^ population that also highly expressed α6 integrin could be manually gated at about ∼5% of the whole cell population (n = 25). ‘Excluded’ cells are 3T3 mouse fibroblasts remaining from the feeder layer used in the culture process. (D) The four populations identified in (C) were separated using FACS and grown *in vitro* for 7 days to analyse colony morphology; H = holoclone-like, M = meroclone-like and P = paraclone-like. (E) Cultured keratinocytes were separated into two populations based upon high α6 integrin and CD44 expression (α6 integrin^high+^/CD44^+^) and the remainder of the cell population (a total of the α6 integrin^+^, CD44^+^, and α6 integrin^−/^CD44^−^ fractions). Simultaneously, keratinocytes were fractionated on the basis of β1 integrin expression (β1 integrin^high^) or α6 integrin and transferrin receptor (α6 integrin^bri^/CD71^dim^) and the corresponding remainder populations (β1 integrin^low^ and α6 integrin^dim^/CD71^bri^). The total cell output from the cells of each fraction was calculated after serial culture *in vitro* until exhaustion (average ∼ 42 days, n = 3). Data represents mean cell yield ± SEM of three replicates. Statistical significance was determined using the paired Student’s *t*-test, *P<0.05. (F) Positive expression of β1 integrin (CD29) and low expression of the transferrin receptor (CD71^dim^) were analysed in the α6 integrin^high+^/CD44^+^ fraction using flow cytometry. Antibody fluorescence is depicted as shaded histograms and the relevant isotype controls as unshaded histograms (n = 3).

In line with previous studies, expression of CD44 was observed in both the basal and suprabasal layers of the human epidermis *in vivo* [13] while α6 integrin was restricted to the basal layer only [4] (Figure 1A). Quantification by flow cytometry of the *in vitro* expression of CD44 and α6 integrin was detected at 10% and 30% of the total human keratinocyte population respectively (Figure 1B). Using two-colour flow cytometry a manual gate was set to analyse basal, α6 integrin-expressing cells (α6 integrin^high+^) that also expressed CD44 (CD44^+^). This fraction (α6 integrin^high+^/CD44^+^) averaged ∼5% of the total keratinocyte population (Figure 1C) and when plated in culture these cells grew to produce large colonies with a tightly-packed holoclone-like morphology (Figure 1D). In contrast, the α6 integrin^−/^CD44^−^ fraction produced only very small, sparse paraclone-like colonies in culture with many keratinocytes remaining as large, single cells indicative of a more differentiated morphology. Whilst both the α6 integrin^+/^CD44^−^ and α6 integrin^−/^CD44^+^ fractions appeared to mainly form meroclone-like colonies.

To establish if there were measurable differences in the long-term growth of the α6 integrin^high+^/CD44^+^ cells compared to the rest of the keratinocyte population, the total cell output was quantified (Figure 1E). When passaged until exhaustion, each α6 integrin^high+^/CD44^+^ cell produced a significantly greater cell output than the remainder population and the UF control (p<0.05). In parallel, the recognised keratinocyte stem cell phenotypes β1 integrin^+^ [3] and α6 integrin^bri^/CD71^ldim^ [5], together with their relative remaining populations (here deemed β1 integrin^−^ and α6 integrin^dim^/CD71^bri^), were run as controls. As expected, both the β1 integrin^high^ and the α6 integrin^bri^/CD71^dim^ fractions produced a larger cell output than their corresponding remainder fractions and the UF cells, although these differences were not significant (p>0.05). The level of β1 integrin^+^ and CD71^dim^ expression in the α6 integrin^high+^/CD44^+^ fraction was quantified at 57% and 42% respectively (Figure 1F).

### α6 Integrin^high+^/CD44^+^ Keratinocytes Display a Resistance to UVB-induced Apoptosis

**Figure 2:**
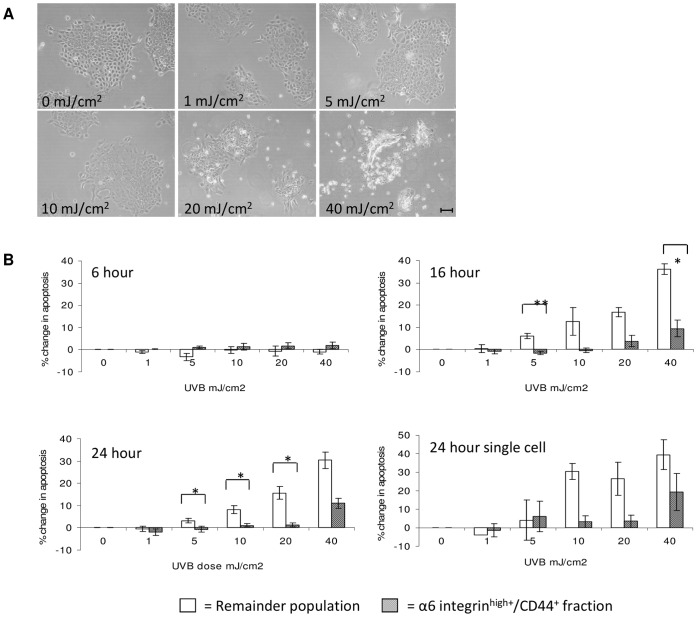
UVB-induced apoptosis. (A) Keratinocytes were grown in culture to form large colonies and photographed 24 hours following UVB irradiation. (B) Quantification of apoptotic cells using flow cytometry. Annexin-V^+^ cells (DAPI^+^ and DAPI^−^) were totalled in both α6 integrin^high+^/CD44^+^ and remainder populations and plotted as a change in percentage from the control level (0 mJ/cm^2^ which averaged ∼8% apoptosis) to negate the necrotic cells present before treatment. Results show apoptosis levels 6, 16 and 24 hours after UVB irradiation of colonies at 50% confluency. Keratinocytes were also UVB irradiated as single cells 2 hours after plating, and apoptosis levels are shown after 24 hours. Data represents the mean ±SEM where n = 3. Statistical significance was determined using the paired Student’s *t*-test **P<0.01, *P<0.05.

Cultured keratinocytes exposed to low doses of UVB (≤10 mJ/cm^2^) revealed very little morphological change at 24 hr post-irradiation compared to control cells (Figure 2A). Keratinocytes exposed to higher doses (>10 mJ/cm^2^) displayed apoptotic damage including membrane blebbing, punctated nuclei and cells lifting from the dish. The change in the early apoptotic marker Annexin-V was compared between the α6 integrin^high+^/CD44^+^ cells and remainder keratinocytes using flow cytometry quantification. At 6 hours following UVB irradiation, no significant apoptotic changes were evident in either fraction (p>0.05, Figure 2B). At 16 and 24 hours post-UVB the remainder population displayed a dose-dependent increase in the proportion of apoptotic cells. In contrast, the α6 integrin^high+^/CD44^+^ fraction displayed a significantly lower level of apoptosis (p<0.05) at doses 5 mJ/cm^2^ and above. Repetition of this assay using keratinocytes plated as single cells prior to UVB irradiation showed a similar pattern of resistance to apoptotic death in the α6 integrin^high+^/CD44^+^ fraction compared to the remainder population (p>0.05 at 24 hr).

### α6 Integrin^high+^/CD44^+^ Keratinocytes Display a Differing Apoptotic Resistance to Genotoxic Agents

**Figure 3:**
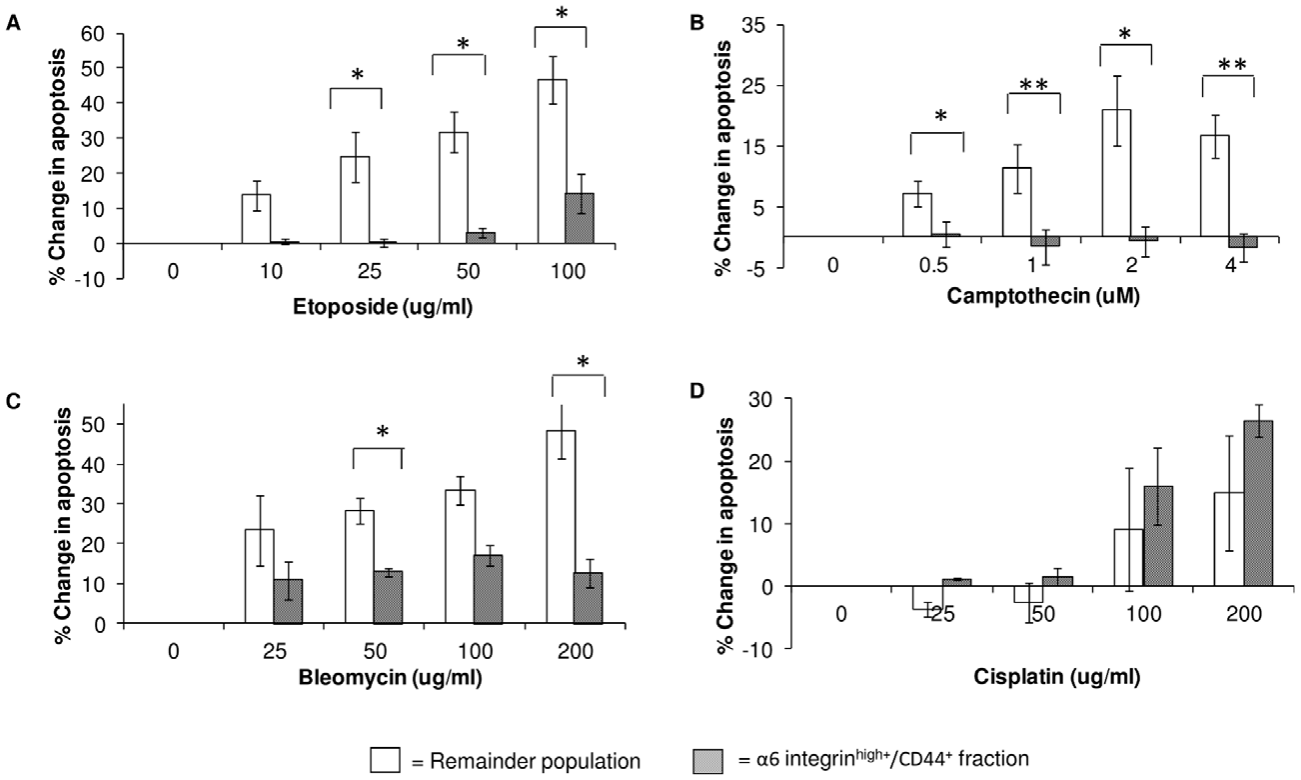
Drug-induced apoptosis. Keratinocytes at 50% confluency treated were with genotoxic agents at increasing doses and analysed for apoptotic cells using flow cytometry. Annexin-V^+^ cells (DAPI^+^ and DAPI^−^) were totalled in both the α6 integrin^high+^/CD44^+^ and remainder cell populations and plotted as a change in percentage from the control (vehicle only control that averaged ∼10% apoptosis). Results show apoptosis levels 24 hours after treatment with Etoposide (A), Camptothecin (B) Bleomycin (C) and Cisplatin (D). Data represents the mean ±SEM where n = 3. Statistical significance was determined using the paired Student’s *t*-test **P<0.01, *P<0.05.

The chemotherapeutic agents etoposide and camptothecin are classified as Topoisomerase inhibitors. Etoposide binds to Topoisomerase II causing both single and double strand DNA breaks [18] and camptothecin binds to Topoisomerase I leading to replication-induced double strand breaks [19]. When increasing doses of the genotoxic agents (etoposide; 10 to 100 µg/ml and camptothecin; 0.5 µM to 4 µM) were added to cultured keratinocytes, the resultant Annexin-V quantification at 24 hr using flow cytometry produced similar patterns to that obtained post-UVB irradiation. A dose-dependent rise in apoptosis was observed in the remainder population, yet the α6 integrin^high+^/CD44^+^ fraction showed significant resistance to apoptosis (p<0.05, Figure 3A and p<0.01, Figure 3B). The antibiotic drug bleomycin cleaves DNA to produce both single and double strand breaks [20]. Addition of bleomycin (35 to 200 µg/ml) produced a similar dose-response pattern in the remainder fraction of increased Annexin-V binding 24 hr post-treatment (Figure 3C). In the α6 integrin^high+^/CD44^+^ fraction an increase in apoptosis was observed above control, but this did not rise in conjunction with increased dose and was still significantly lower than the remainder population (p<0.05).

Treatment with the platinum DNA cross-linking agent cisplatin produced an altered apoptotic response in the keratinocytes. As the concentration of cisplatin (25 to 200 µg/ml) administered to the keratinocytes was increased, the level of Annexin-V binding rose in both the remainder and α6 integrin^high+^/CD44^+^ populations (Figure 3D).

### Cell Death is Apoptotic in Origin

**Figure 4:**
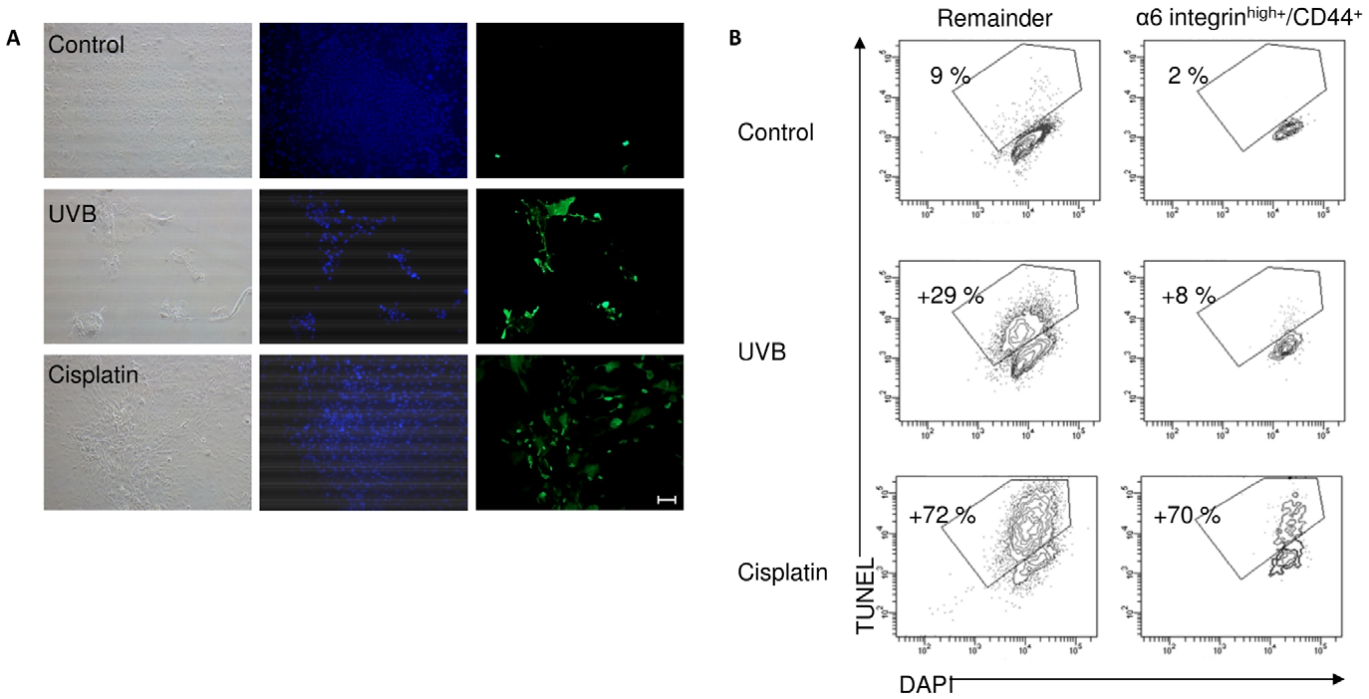
Apoptosis detection using Active Caspase-3 and TUNEL. (A) Keratinocytes were grown for 5 days to form colonies, treated with UVB (20 mJ/cm^2^) or cisplatin (200 µg/ml) or vehicle-only (control). After 24 hr the cells were stained with an antibody against Active Caspase-3 and photographed. Nuclei are stained blue with DAPI. (B) Keratinocytes were grown for 5 days, treated with UVB (30 mJ/cm^2^), cisplatin (50 µg/ml) or vehicle-only (control) for 24 hours, then fixed and analysed for TUNEL^+^ cells using flow cytometry. Data represents the mean where n = 3.

Immunofluorescence staining to visualise the later-stage apoptotic marker of active Caspase-3 revealed a greater number of active Caspase-3 positive cells were present at 48 hr in keratinocytes treated with either UVB or cisplatin compared to untreated control cells (Figure 4A).

Flow cytometry was used to analyse the number of TUNEL^+^ cells in the last stage of programmed cell death, 24 hr after UVB or cisplatin treatment. The change in apoptotic DNA fragmentation from the control was significantly higher in the remainder fraction than the α6 integrin^high+^/CD44^+^ fraction after UVB irradiation (p<0.05, Figure 4B). Whereas the number of cells in the last stage of programmed cell death was very similar in both fractions following cisplatin treatment (p>0.05).

**Figure 5:**
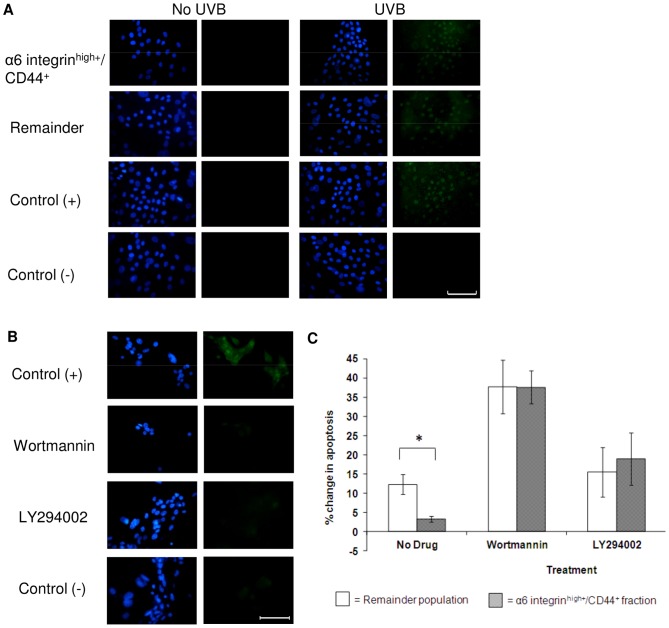
Apoptosis following inhibition of the PI-3 Kinase Pathway. (A) Immunofluorescence detection of phosphorylated Akt (pAkt^Ser473^) in FACS-separated α6 integrin^high+^/CD44^+^ and remainder keratinocytes treated with 30 mJ/cm^2^ UVB irradiation. The positive control is the unfractionated total keratinocyte population probed with both the primary and secondary antibodies and the negative control prepared with the secondary antibody only. Images are representative of n = 3 and cell nuclei were detected with DAPI (blue). The scale bar is equal to 100 µm. (B) Sub-confluent primary keratinocytes probed for detection of phosphorylated Akt (pAkt^Ser473^) following treatment with 1 µM wortmannin or 50 µM LY294002 prior to UVB irradiation (30 mJ/cm^2^). Control cells were treated with the vehicle-only and negative controls prepared by omission of the primary antibody. Cell nuclei were detected using DAPI (blue) and images are representative of n = 3. The scale bar is equal to 100 µm. (C) Primary keratinocytes treated with 1 µM Wortmannin, 50 µM LY294002 for 2 hr, or no drug (vehicle-only control), were analysed for positive TUNEL expression 24 hr post-irradiation with UVB (30 mJ/cm^2^). The graph shows TUNEL^+^ cells in the α6 integrin^high+^/CD44^+^ and remainder populations plotted as a change in percentage from control level (0 mJ/cm^2^, ∼ 3.5% apoptosis). Data represents the mean ± SEM where n = 4. Statistical significance was determined using the paired Student’s t-test, *P<0.05.

### Inhibition of the PI 3-kinase Survival Pathway Sensitises the α6 Integrin^high+^/CD44^+^ Keratinocytes to UVB-induced Apoptosis

To identify if the PI3-kinase cell survival pathway is protecting the α6 integrin^high+^/CD44^+^ fraction, the cells were probed for expression of the activated downstream effector pAKT. An increase in positive nuclear expression of pAkt^Ser473^ was observed following UVB irradiation, but no difference between the fractions could be identified using immunofluorescence (Figure 5A). No positive nuclear expression of pAkt^Ser473^ was observed post-UVB when the PI 3-kinase pathway had been inhibited using Wortmannin or LY294002 prior to irradiation (Figure 5B). PI3-kinase pathway inhibition using Wortmannin or LY294002 sensitised the α6 integrin^high+^/CD44^+^ keratinocytes to apoptotic cell death. The rise in TUNEL^+^ cells was comparable between the α6 integrin^high+^/CD44^+^ cells and the remainder fraction when quantified by flow cytometry (p>0.05, Figure 5C).

**Figure 6:**
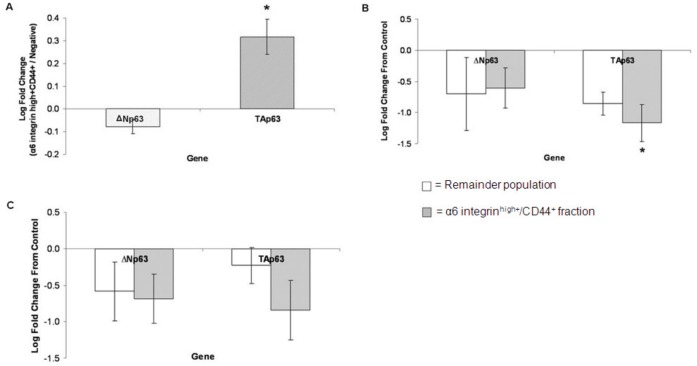
p63 gene expression. (A) Primary keratinocytes analysed for p63 gene expression in the α6 integrin^high+^/CD44^+^ and remainder populations using QRT-PCR. Data represents the log mean fold change of the α6 integrin^high+^/CD44^+^ population from the remainder population ± SEM of three replicates, normalised against GAPDH housekeeping gene. Keratinocytes analysed for gene expression after cisplatin (B) or UVB (C) where data represents the log mean fold change from the control (vehicle-only) ± SEM of three replicates, normalised against 28S-r-RNA housekeeping gene. Statistical significance was determined using the paired Student’s t-test, *P<0.05.

### p63/c-abl Network Activated in Cisplatin-induced Cell Death in α6 Integrin^high+^/CD44^+^ Keratinocytes

The full length TAp63 isoform of the proposed keratinocyte stem cell marker p63 [21], itself identified as an initiator of apoptosis [22], was found to be expressed at a significantly higher mRNA level in the α6 integrin^high+^/CD44^+^ cells compared to the remainder population when quantified using QPCR (p<0.05, Figure 6A). Gene expression of the truncated ΔNp63 isoform did not differ between the two keratinocyte fractions. Cisplatin treatment appeared to reduce the gene expression levels of the ΔNp63 isoform, with little difference observed between the α6 integrin^high+^/CD44^+^ and remainder populations (p>0.05, Figure 6B). mRNA levels of the TAp63 isoform also reduced following cisplatin treatment, with the α6 integrin^high+^/CD44^+^ cells displaying a significant decrease from the control level (p<0.05). Following UVB irradiation, the levels of both p63 isoforms decreased slightly in both populations (p>0.05, Figure 6C).

**Figure 7:**
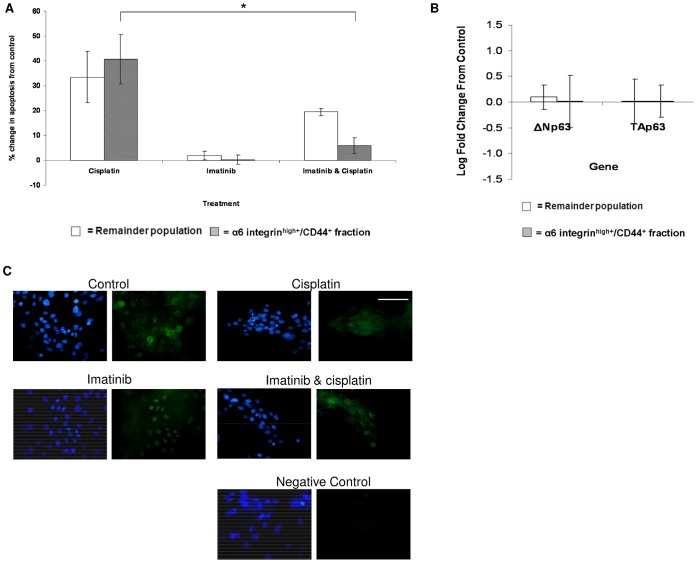
Effect of imatinib on keratinocyte apoptosis and p63 expression. (A) Primary keratinocytes treated with 50 µg/ml cisplatin, or 1 µM imatinib, or 1 µM imatinib for 2 hr and then 50 µg/ml cisplatin were analysed for TUNEL expression 24 hour post-treatment. TUNEL-positive cells were totalled in the α6 integrin^high+^/CD44^+^ and remainder populations and plotted as a percentage change from control level. Data represents the mean ± SEM where n = 3. Statistical significance was determined using the paired Student’s t-test, *P<0.05.) (B) QRT-PCR quantification of p63 gene expression in α6 integrin^high+^/CD44^+^ and remainder keratinocyte populations 16 hr after treatment with 1 µM imatinib and 50 µg/ml cisplatin. Data represents the log mean fold change from the control (vehicle-only) for each population ± SEM of three replicates. All results were normalised against the 28s-r-RNA housekeeping gene. Statistical significance was determined using the paired Student’s t-test (P>0.05 in all cases). (C) FACS-separated α6 integrin^high+^/CD44^+^ cultured keratinocytes were probed for p63 expression (green) using immunofluorescence 16 hr following treatment with 50 µg/ml cisplatin and/or 1 µM imatinib. The control cells were treated with vehicle-only and the negative control was prepared with the secondary antibody only. Nuclei were detected with DAPI (blue) and images are representative of n = 2. The scale bar is equal to 100 µm.

Addition of the tyrosine kinase inhibitor imatinib prior to cisplatin treatment significantly reduced the amount of cell death seen in the α6 integrin^high+^/CD44^+^ keratinocytes compared to treatment with cisplatin alone (p<0.05, Figure 7A). Whilst the remainder population did display a decrease in the level of apoptosis, this was not as marked as that seen in the α6 integrin^high+^/CD44^+^ keratinocytes, nor significant (p>0.05).

Gene expression of the ΔNp63 and TAp63 isoforms did not alter from the control (vehicle-only) level in the remainder keratinocyte population when treated with imatinib prior to cisplatin (Figure 7B). The significantly higher level of TAp63 mRNA quantified in the α6 integrin^high+^/CD44^+^ fraction did not decrease in response to cisplatin with imatinib. This is in contrast to the decrease in the gene expression of this isoform reported in the cisplatin-only treatment.

Analysis of the location of the total p63 protein in the α6 integrin^high+^/CD44^+^ keratinocytes revealed positive nuclear expression evident in the control cells (Figure 7C). The positive nuclear expression was lost when the cultured cells were treated with cisplatin, yet regained with the addition of imatinib prior to cisplatin. This pattern was repeated in the remainder population of keratinocytes.

## Discussion

Here we report the identification of a small basal fraction of cultured human primary keratinocytes that express CD44 and display a high growth capacity coupled with an enhanced resistance to apoptosis induced by UVB and specific DNA damaging agents.

Separation of cultured cells expressing high levels of the adhesion molecule α6 integrin enabled selection of the less-differentiated basal keratinocyte population. Analysis of the integrin^high+^ cells for positive CD44 expression revealed a co-expressing population that could be manually gated at ∼5% of the total keratinocyte population (α6 integrin^high+^/CD44^+^ cells). The morphology of the colonies formed in culture by this fraction of cells were predominantly holoclone-like in appearance and when passaged until exhaustion, the total cell output was of an equivalent level to that produced by recognised KSC phenotypes β1 integrin^high^ and α6 integrin^bri^/CD71^dim^. When directly analysed for β1 integrin expression or low levels of CD71, the α6 integrin^high+^/CD44^+^ fraction contained a large number of cells previously identified as KSCs as well as other un-differentiated transient-amplifying cells.

To determine if this population displayed a different apoptotic capability compared to the rest of the cell population, the response of the cells to UVB irradiation was explored. The α6 integrin^high+^/CD44^+^ fraction displayed a significantly decreased level of apoptosis compared to the rest of the keratinocytes. Interestingly, α6 integrin has also been shown to be required for the growth and survival of breast cancer cells [23], and an increased expression of CD44 has been observed in cells displaying an apoptotic resistance, in line with a decreased p53 expression [22] or an increased p63 expression [24]. Evaluation of the p63 mRNA levels in our α6 integrin^high+^/CD44^+^ keratinocytes produced surprising results. QRT-PCR quantification demonstrated a significantly higher mRNA level of the TAp63 isoform, rather than the ΔNp63 isoform, present in the α6 integrin^high+^/CD44^+^ cells compared to the remainder population (p<0.05). This was unexpected due to the α6 integrin^high+^/CD44^+^ phenotype being selected on the basis that these were the CD44-expressing cells co-expressing the basal layer marker α6 integrin. The truncated form of p63 (ΔNp63), lacking the NH2-terminal transactivation domain of the full length TAp63 isoform, has repeatedly been identified as the predominant isoform of p63 detected in the basal layer of the human and the murine epidermis [25] and has also been proposed as an inhibitor of apoptosis in the epidermis [26]. This isoform has also been shown to induce integrin expression in murine keratinocytes [27]. TAp63 has been found at only very low levels in the epidermis and in isolated keratinocytes [25] and was identified as an initiator of apoptosis in a wide range of tissues [26]. However, in line with our findings, researchers have begun to question the proposed apoptosis-associated role of TAp63 in the epidermis. Analysis of p63 expression in mice during gestation revealed that TAp63 is actually the dominant isoform present from E7.5 to E15 [28]. Interestingly, overexpression of TAp63, rather than ΔNp63, leads to a significant reduction in the level of apoptosis after UVB irradiation in murine keratinocytes [29]. Analysis of the TAp63 mRNA after UVB irradiation in our α6 integrin^high+^/CD44^+^ human keratinocytes showed a slight decrease in the gene expression level, but was not significant. The lack of any rise could demonstrate that TAp63 is not taking on a pro-apoptotic role in the keratinocytes and the lack of significant decrease could infer that it is the high basal level of TAp63 that is offering resistance to UVB-induced apoptosis in this human population. This is in agreement with the finding in the murine keratinocytes that the basal level of TAp63 infers a resistance to UVB-induced apoptosis. Apoptosis is augmented following a loss of the basal TAp63 through siRNA knockdown of the protein [29]. In the murine keratinocytes, inhibition of UVB-induced apoptosis was linked to an increase in phosphorylation of the cell survival protein Akt (pAkt^Ser473^), induced by the increased TAp63 expression. It was confirmed in this study that the PI 3-Kinase pathway plays a role in the human α6 integrin^high+^/CD44^+^ apoptotic resistance, for the survival of the cells was abolished when inhibitors of the PI 3-Kinase pathway were added prior to UVB irradiation. The inhibitors rendered the α6 integrin^high+^/CD44^+^ keratinocytes as sensitive to UVB-induced apoptosis as the rest of the cultured cell population. It was also confirmed that addition of these inhibitors did not lead to the increase in nuclear pAkt^Ser473^ expression observed following UVB irradiation-alone.

The α6 integrin^high+^/CD44^+^ keratinocytes were also significantly resistant to DNA damage induced by the chemotherapeutic agents etoposide and camptothecin. The striking sensitivity to cisplatin was in contrast to the resistance noted in epithelial cancer stem cell lines [12]. Recent research conducted in mouse oocytes had established the presence of a cisplatin-induced pathway of apoptosis involving the tyrosine kinase c-abl. In particular, the dominance of the role of TAp63 was in activating this c-abl-pathway was demonstrated [30]. However, when p63 mRNA levels were examined in the α6 integrin^high+^/CD44^+^ cells following cisplatin treatment, a significant decrease in TAp63 was observed. This implies that the loss of the basal level of TAp63 has reduced the associated resistance of the cells, as opposed to an increase that could infer a TAp63/c-abl apoptotic pathway is present. In accordance, a loss of nuclear p63 protein expression was observed following cisplatin treatment of the cultured cells rather than a stabilisation, or increase, in protein expression via c-abl activation following cisplatin-induced DNA damage [30]. Interestingly, addition of the tyrosine kinase inhibitor imatinib caused the resistance of the α6 integrin^high+^/CD44^+^ to be increased, so that the cells were significantly resistant to cisplatin-induced apoptosis compared to the rest of the population. This finding indicates the presence of a specific cisplatin-activated tyrosine kinase is able to overcome the basal resistance of the cells seen in response to other genotoxic agents. This basal resistance is again linked back to p63, with the observation that the p63 protein is returned to the control-location of the nucleus when imatinib is added prior to cisplatin treatment.

In conclusion, we have identified a sub-population of cultured human keratinocytes expressing high levels of CD44 and α6 integrin that display a high growth capacity. These α6 integrin^high+^/CD44^+^ cells display a resistance to undergo programmed cell death in response to UVB irradiation and chemotherapeutic drugs, except in the case of cisplatin treatment where a sensitivity to undergo apoptosis in line with the rest of the cell population is observed. The resistance to UVB-induced apoptosis could be an important property that allows the regenerative capacity of the undifferentiated cells in the human skin to be maintained despite acute UVB-exposure. The mechanism of this resistance appears to be attributed to a high level of baseline TAp63 gene expression linked to the PI 3-Kinase cell survival pathway. The contrasting sensitivity to cisplatin treatment appears to be due to the ability of the drug to cause a loss of this TAp63-conferred protection. This loss is likely to be due to the cisplatin-induced activation of a tyrosine kinase causing a reduction in TAp63 gene expression and nuclear p63 protein levels. Addition of a tyrosine kinase inhibitor reduces the apoptotic sensitivity of the cells to cisplatin, so they are able to demonstrate a resistance to the platinum agent-induced apoptosis. Correspondingly, the high baseline TAp63 mRNA expression does not decrease and the nuclear expression of the p63 protein is not lost.

The notion that a network of TAp63- linked apoptotic protection is present in a population of undifferentiated human keratinocytes may provide important information regarding the behaviour of long-lived cells in the human epidermis. If the resistance to undergo cell death following UVB irradiation is repeated *in vivo*, the keratinocyte sub-population identified here could provide evidence regarding the PI 3-Kinase regulated cell survival. The possibility that proliferative cells could be retaining UVB-induced mutations and undergoing subsequent transformation would link in with the evidence that epidermal squamous cell carcinomas (SCCs) routinely display a high level of p63 expression. Correspondingly, the finding that these cells are particularly sensitive to cisplatin could provide evidence on how to target and treat the population with chemotherapeutic agents.
